# Comment on “Does the position of shoulder immobilization after reduced anterior glenohumeral dislocation affect coaptation of a Bankart lesion? An arthrographic comparison.” by Momenzadeh OR et al.

**DOI:** 10.1007/s10195-015-0382-7

**Published:** 2015-10-22

**Authors:** John W. Sperling

**Affiliations:** Department of Orthopedic Surgery, Mayo Clinic, Rochester, MN USA

The authors present a very interesting article [1] that compares the position of the arm in immobilization after anterior dislocation. Anterior glenohumeral instability is a common problem, and therefore this study has the potential for significant and widespread impact. In the past, anterior dislocations were routinely treated with a sling and the arm in an internally rotated position. There have been a few reports noting the potential for improved healing of the Bankart lesion if the arm is placed in external rotation. However, there has been minimal magnetic resonance imaging data on the true anatomic impact of different immobilization positions.

The investigators are to be congratulated on a well-organized study. The results of this investigation clearly demonstrate that the externally rotated group had decreased separation, displacement, and opening angle compared to the internally rotated group. One caveat of the study is that some patients may have discomfort with the externally rotated position and may not be able to tolerate this treatment. Overall the methodology is very sound and the results are important for those treating patients with shoulder disorders.
